# Epidemiology of hypertension among Bangladeshi adults using the 2017 ACC/AHA Hypertension Clinical Practice Guidelines and Joint National Committee 7 Guidelines

**DOI:** 10.1038/s41371-018-0087-5

**Published:** 2018-07-19

**Authors:** Jessica Y. Islam, M. Mostafa Zaman, Syed Atiqul Haq, Shamim Ahmed, Zahid Al- Quadir

**Affiliations:** 1 Department of Epidemiology, Gillings School of Global Public Health, UNC Chapel Hill, Chapel Hill, NC USA; 2 World Health Organization, Dhaka, Bangladesh; 3 Department of Rheumatology, Bangabandhu Sheikh Mujib Medical University, Shahbag, Dhaka, Bangladesh

## Abstract

In 2017, the American College of Cardiology (ACC) and American Heart Association (AHA) released updated guidelines on the definition of hypertension, and blood pressure thresholds for initiation of antihypertensive medication. Our objective was to assess the change in prevalence of hypertension, risk factors, and identify populations recommended for treatment among Bangladeshi adults, based on the 2017 ACC/AHA guidelines. Data for this analysis were collected from a population-based nationally representative sample of 1843 Bangladeshi adults, aged ≥18 years in 2015. Hypertension was defined based on two definitions: the JNC 7 guidelines (SBP ≥ 140 or DBP ≥ 90 mmHg), and the 2017 ACC/AHA guidelines (SBP ≥ 130 mmHg, or DBP ≥ 80 mmHg), or a self-reported diagnosis of hypertension. Based on the 2017 ACC/AHA guidelines, the prevalence of hypertension was 40.7% (95% CI: 38.5–43.0). The prevalence of JNC 7 definition of hypertension was 17.9% (95% CI: 16.2–19.7), indicating a 22.8% increase in prevalence. Based on both definitions, urban residents, older adults, adults with low physical activity, obese, abdominally obese, and diabetic adults were more likely to have hypertension. Based on current JNC 7 guidelines, only half of hypertensive adults were aware of having hypertension. Among those aware of their condition, 75% were taking medication based on self-report. Using the 2017 ACC/AHA guidelines, the prevalence of hypertension will more than double in Bangladesh. Newly diagnosed hypertensive adults will be considered high-risk for cardiovascular disease leading to a larger burden on Bangladesh’s health system. However, implementation of the ACC/AHA guidelines may improve prevention efforts where lifestyle changes are appropriate.

## Introduction

Globally, cardiovascular diseases (CVDs) lead to one-third of all annual deaths, which is nearly 17 million total deaths worldwide [1]. Of these deaths due to CVDs, complications of hypertension account for 53% of CVD-related mortality worldwide every year. Although highly preventable, hypertension is the most prevalent risk factor for CVD [2, 3]. The global prevalence of hypertension is projected to increase from 26% in 2000 to 29.2% by 2025 [3]. Factors attributed to the increase in prevalence of hypertension include, population growth, ageing of the population, and behavioral risk factors, such as smoking, poor diet, harmful use of alcohol, low physical activity, and overweight or obesity [1]. Globally, the observed increasing burden of hypertension is a major public health concern.

In 2017, the American College of Cardiology and the American Heart Association (ACC/AHA) published updated guidelines titled, The ACC/AHA Guidelines for the Prevention, Detection, Evaluation and Management of High Blood Pressure in Adults or the 2017 Hypertension Clinical Practice Guidelines [4]. These guidelines provide updated recommendations for the diagnostic criteria of hypertension, appropriate blood pressure (BP) criteria for pharmacologic intervention, BP target goals, diagnostic work and evaluation for hypertension, and lifestyle management strategies both for prevention and for treatment of hypertension [5]. The 2017 ACC/AHA guidelines were developed based on evidence from recently published randomized control trials, and a broader range of evidence, such as epidemiological studies and expert opinion [5, 6]. However, of note, several scientific organizations, such as the American College of Physicians and the American Academy of Family Physicians, have not endorsed the updated guidelines particularly its applicability to those aged 60 years and above [7]. This may impact uptake of the updated diagnostic criteria of hypertension and recommendations for care and treatment, included in the 2017 ACC/AHA among physicians in the United States.

Although the guidelines were developed with a focus on medical practice in the United States [6], universal implementation of the new guidelines may lead to a change in prevalence of hypertension on a global scale [8]. If fully or partially adopted, the 2017 ACC/AHA guidelines may have implications on treatment and prevention of the development of CVDs globally. Here we present the prevalence of hypertension in Bangladesh, a nation with high CVD-related morbidity and mortality [9–12], using the 2017 ACC/AHA. Our primary aim was to evaluate the change in prevalence of hypertension based on the 2017 ACC/AHA in comparison to the AHA guidelines followed previously, called the Joint National Committee 7 Blood Pressure Guidelines or JNC 7. Previous papers have found poor medication adherence and uptake of treatment for hypertension among Bangladeshi adults [13–16]. As such,  our secondary aim was to compare the proportion of participants who self-reported to take medication to control their hypertension, to those recommended for antihypertensive medication based on the 2017 ACC/AHA updated guidelines. Data from this novel analysis will inform public health interventions in Bangladesh to address the growing burden of hypertension and subsequent CVD.

## Methods

For this analysis, data were obtained from a national survey to assess the burden of musculoskeletal disorders in Bangladesh. This national survey was conducted with technical support of the World Health Organization Country Office for Bangladesh and Bangabandhu Sheikh Mujib Medical University (BSMMU) [17]. Conducted from November to December 2015, the study was a population-based cross-sectional research design, and followed the WHO STEP-wise approach to Surveillance of NCD risk factors (STEPS) [18]. Men and women aged 18 years and above residing in rural and urban areas of Bangladesh were included. The exclusion criteria of this survey included, tourists and the institutionalized. Throughout the study, ethical guidelines outlined by the Declaration of Helsinki were followed and ethical clearance was obtained from BSMMU Internal Review Board. We obtained written consent from participants in Bangla as per BSMMU guidelines. Participants unable to write provided thumb impressions as part of the consent process.

### Sampling methods

To obtain a nationally representative sample of Bangladesh, the study adopted a multistage, geographically clustered, probability-based sampling approach. Population statistics were obtained using the national census conducted by Bangladesh Bureau of Statistics (BBS) [19]. Bangladesh is divided into seven divisions. Each division is divided into several districts (*Zila*) and subdistricts (*Upazila*). Within subdistricts, *mauzas* and *mahallas* are the smallest units within defined territories in rural and urban areas, respectively. *Mauzas* and *mahallas* were considered the primary sampling unit (PSU) for this sampling approach. The households within the *mauzas* and *mahallas* were the secondary sampling units. We defined a household as “a dwelling in which persons either related or unrelated were living together and using the same kitchen” [19].

The power analysis and sample size calculations were completed based on the standardized approach outlined in WHO STEPS methodology [18]. We estimated the minimum number of participants required was 296 in each group (rural males, rural females, urban males, and urban females). Assuming a design effect of 1.5 adjusted within cluster population homogeneity, the necessary sample size was 1774. Our target sample size was 2000, as we assumed about 90% response rate. Twenty PSUs (8 urban and 12 rural) were randomly selected from 7 divisions of the country, with the probability proportional to the population size of each division. In each PSU, 100 consecutive households were selected. The even numbered households were designated as a “male household” and odd numbered households as a “female household.” Finally, one male or female was approached to participate from each respective household as designated.

### Data collection

The survey instrument collected information on the following topics: musculoskeletal disorders [15], and demographic data such as age, area of residence, education, current (last 12 months) occupation, tobacco use, and physical activity. Physical measurements such as height, weight, waist circumference, blood glucose levels, and BP were collected. Participants were asked if they had been previously diagnosed by a health care provider with high BP or diabetes based on self-report. Additionally, participants were asked if they were taking medications to control their diagnosed condition. Specifically, participants were asked: 1. Have you ever been diagnosed with high BP by a health care professional? and, 2. If yes, are you receiving treatment for high BP. We were unable to assess history of cardiovascular disease or take blood samples for lipid measurements. The questionnaire was translated from English to Bengali, adapted, and validated as per standard procedure.

### BP measurement

Methods used for BP measurement have been previously described [16]. In summary, BP was measured using an appropriately calibrated aneroid sphygmomanometer and arm cuffs. The initial measurement was performed after 5 min of rest on the right arm. After 2 min, the second measurement was taken. We used the average of the two BP readings as each participant’s final BP measurement.

### Definition of hypertension and recommendations for antihypertensive medication

Our primary outcome of interest was the prevalence of hypertension. Based on the 2017 ACC/AHA, diagnostic criteria for hypertension was systolic blood pressure (SBP) of ≥130 mmHg (millimeters of mercury) and/or, diastolic blood pressure (DBP) of ≥80 mmHg. To evaluate the change in prevalence of hypertension, we calculated the prevalence of hypertension using the JNC 7 guidelines. Based on the JNC 7, diagnostic criteria for hypertension was SBP of ≥140 mmHg and/or, DBP ≥90 mmHg. Additionally, those who self-reported to be taking anti-hypertensive medication were included in the JNC 7 definition of hypertension.

We present stages of hypertension as defined by the 2017 ACC/AHA, including normal BP, elevated BP, hypertension stage 1, and hypertension stage 2 [4]. To evaluate the prevalence of hypertension by stage, individuals who self-reported to take anti-hypertensive medication were included only in hypertension stage 2. We calculated the number of adults recommended to take antihypertensive medication based on the 2017 ACC/AHA guidelines using the following categories: (1) general population with a SBP ≥ 140 mmHg or DBP ≥ 90 mmHg; (2) individuals with diabetes ≥ 130 mmHg or DBP ≥ 80 mmHg; and (3) those aged ≥ 65 years with SBP ≥ 130 mmHg. The 2017 ACC/AHA guidelines also recommend antihypertensive medications for those with high cardiovascular risk [20]. However, we were unable to include this in our analysis as we did not record history of CVD in our survey.

### Covariates

The following variables were used as covariates for analyses: area of residence, sex, age, education, occupation, wealth index, body mass index (BMI), blood glucose levels, and waist circumference. Age was categorized into four age groups: 18–29, 30–44, 45–59 and ≥60 years. We categorized education into four groups: no education, primary education (completed grade 5 and below), secondary education (completed grade 10 and below), and above secondary education (completed grade 12 and above). Occupation was categorized into five groups for this analysis. Data on physical activity was collected based on self-report per STEPS protocol [21]. Physical activity was then categorized based on total MET-minutes. Participants who spent 3000 or more MET-minutes per week were categorized to have vigorous physical activity group, 600–3000 MET-minutes were categorized in moderate physical activities, and below 600 MET-minutes were categorized to have low physical activity [22]. The wealth index was constructed using principal component analysis. Further details describing how the wealth index was calculated can be found in a prior publication [16].

To calculate BMI, we used the height (in centimeters) and weight (in kilograms) measurements. We categorized BMI to the following groups: underweight (≤18.50), normal (18.6–25), overweight (25.1–30), and obese (>30). Waist circumference was measured in centimeters (cm). Participants were categorized as abdominally obese if waist circumference was 90 cm and above for males, or 80 cm and above for females. Participants were categorized to have diabetes if their random plasma glucose level was ≥11.1 mmol/L or if they self-reported to take medication to control their diabetes.

### Data analysis

We present sociodemographic variables using median (interquartile range) for continuous variables and proportions for categorical variables. We conducted bivariate analyses by sex and age group to assess differences in hypertension prevalence. We performed chi-square tests to assess for potential differences in hypertension across select demographic variables. We performed unadjusted and adjusted logistic regression analyses to identify significant predictors of hypertension based on the two different guideline definitions. To identify potential risk factors of hypertension to be included in the model, we used bivariate logistic regression analysis and an arbitrary *p*-value of <0.10 to assess suitability for inclusion of variables in the multivariable logistic regression model. For multivariable logistic regression models, we calculated adjusted odds ratios (aOR), and 95% confidence intervals (CI) for each independent variable. We assessed collinearity to ensure there were no strong linear relationships among independent variables included in the model. All statistical procedures were performed using Stata/SE 15.0 software package.

## Results

### Demographic characteristics

Overall, 1843 adults from urban (*n* = 716) and rural (*n* = 1127) regions of the country were included in this study. Forty-eight percent (*n* = 892) were male. The median age and education level of participants was 38 (29.50) years and 5 (0.9) years, respectively. The majority of the population was married (88.2%) and employed as either an industrial worker/day laborer (26.2%) or housewife (40.7%). The study population was generally evenly distributed across wealth quartiles, with the highest proportion occurring in the 2nd quartile (29.1%). Almost half of the population (44.8%) reported to use tobacco currently. The median BMI was 21.4 (19–24.5) and median waist circumference was 77.5 (70.1–86.1). Overall, the median SBP was 113 mmHg (104–124) and DBP was 77 mmHg (70–81) (Table 1).

### Prevalence and stages of hypertension

We found the prevalence of hypertension was 17.9% (95% CI: 16.2–19.7), based on the original JNC 7 guidelines. Using the 2017 ACC/AHA guidelines, the prevalence of hypertension among Bangladeshi adults increased to 40.7% (95% CI: 38.5–43.0). This indicated an increase in prevalence by +22.8 percentage points based on the 2017 ACC/AHA compared to JNC 7 guidelines.

**Table 1:** Background characteristics of Bangladeshi adult participants, 2015 (*n* = 1843)

Characteristic	Total (*n* = 1843)			Urban (*n* = 716)			Rural (*n* = 1127)		
	Mean (SD)	*n*	%	Mean (SD)	*n*	%	Mean (SD)	*n*	%
*Gender*									
Male		892	48.4		345	48.2		547	48.5
Female		951	51.6		371	51.8		580	51.4
Age (years)	40.5 (14.7)			39.1 (13.9)			41.4 (15.1)		
Education (years)^a^	5 (0–9)			8 (3–12)			4 (0–8)		
*Marital status*									
Never married		110	5.9		54	7.5		56	4.9
Married		1625	88.2		627	87.6		998	88.6
Separated/divorced/widowed		108	5.9		35	4.9		73	6.5
*Occupation*									
Professional employment^b^		280	15.2		190	26.5		90	7.9
Unemployed/retired		98	5.3		43	6.0		55	4.9
Industrial worker/day laborer		483	26.2		120	16.7		363	32.2
Housewife		749	40.7		254	33.5		495	43.9
Other^c^		232	12.6		109	15.2		123	10.9
*Wealth index* ^d^									
1st Wealth quartile		414	22.5		111	15.5		303	26.9
2nd Wealth quartile		536	29.1		171	23.9		365	32.4
3rd Wealth quartile		436	23.7		183	25.6		253	22.5
4th Wealth quartile		457	24.8		251	35.1		206	18.3
*Tobacco use* ^e^									
Never		878	47.6		397	55.5		481	42.7
Current use		826	44.8		268	37.4		558	49.5
Past use		139	7.5		51	7.1		88	7.8
*Smoking tobacco use* ^f^									
Current use (daily/non-daily)		494	26.8		169	23.6		325	28.8
Past use		104	5.6		38	5.3		66	5.9
Never		1245	67.8		509	71.1		736	65.3
*Smokeless tobacco use* ^g^									
Current use (daily/non-daily)		534	28.9		150	20.9		384	34.1
Past use		51	2.8		21	2.9		30	2.7
Never		1258	68.3		545	76.1		713	63.3
*Physical activity* ^h^									
Vigorous		1278	69.3		448	62.6		830	73.7
Moderate		478	25.9		239	33.4		239	21.2
Low		87	4.7		29	4.1		58	5.2
Body mass index^i^	22.1 (4.1)			23.3 (4.5)			21.3 (3.7)		
Waist circumference (cm)	78.4 (11.6)			81.8 (12.6)			76.2 (10.3)		
*Blood pressure*									
Systolic blood pressure (mmHg)	116.1 (17.1)			117.9 (16.6)			115.0 (17.3)		
Diastolic blood pressure (mmHg)	76.1 (10.5)			77.9 (10.9)			74.9 (10.1)		
Blood blucose level (mmol/l)	6.4 (2.4)			6.6 (2.9)			6.3 (2.1)		

*SD* standard deviation

^a^Calculated median and interquartile range for education as the data are skewed

^b^Professional occupation includes: Field staff, police officer, guard, doctor, engineer, professional, business man, desk job

^c^Other occupation includes: shop keeper, weavers, driver, student, beggar, cook, carpenter, tailor, migrant workers and fishermen

^d^Wealth index was calculated using principal component analysis using data collected on household ownership of the following items: electricity, flushable toilet, land phone, cell phone, television, radio, refrigerator, private car, motor cycle, washing machine, bicycle, sewing machine, almirah/wardrobe, table, bed, chair/bench, watch/clock, as well as, type of main material used to build their homes roof, walls and floor

^e^Includes both smokeless tobacco and smoke tobacco

^f^Smoking tobacco use includes cigarettes, biri, hookah, etc.

^g^Smokeless tobacco use includes jodda, paan, white leaf, etc.

^h^Measured in MET-minutes; 1 MET stands for the amount of oxygen you consume and the number of calories you burn at rest

^i^Body mass index (BMI) calculated by weight in kilogram divided by height in meter squared

**Fig. 1:**
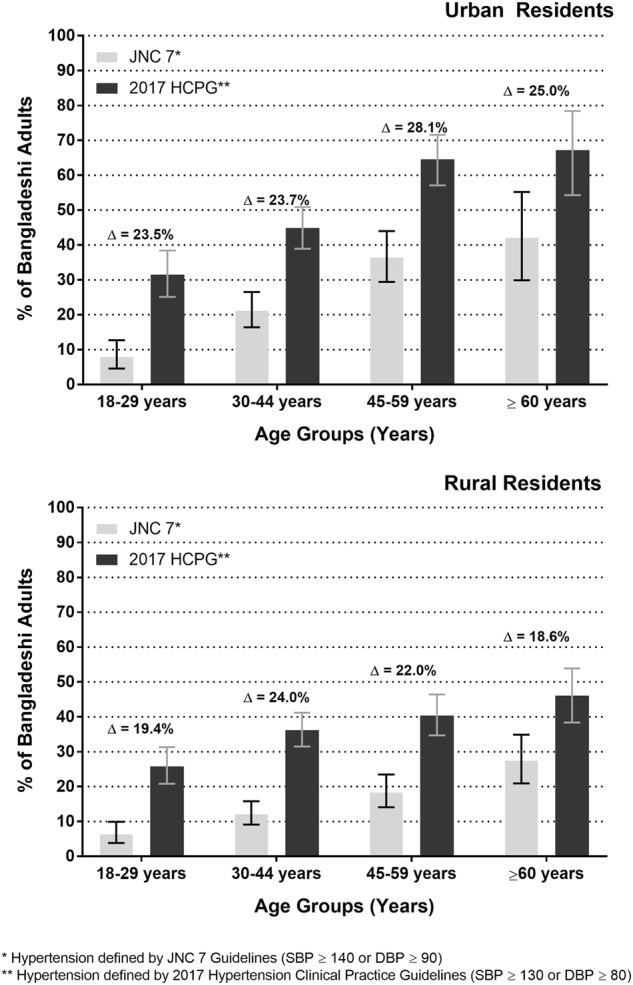
Prevalence and change in prevalence of hypertension based on two SBP/DBP guidelines among Bangladeshi adults aged 18 years and above by area of residence, 2015 (n = 1843)

Figure 1 presents a summary of the change in prevalence of hypertension as defined by the 2017 ACC/AHA and JNC 7 across key demographic variables. Here, we find the prevalence of hypertension increased across age groups among both urban and rural participants. We observed the highest prevalence of hypertension among urban residents aged 45–59 years and 60 years and above. For these groups, the change in prevalence of hypertension was +28.1% and +25.0%, respectively.

**Fig. 2:**
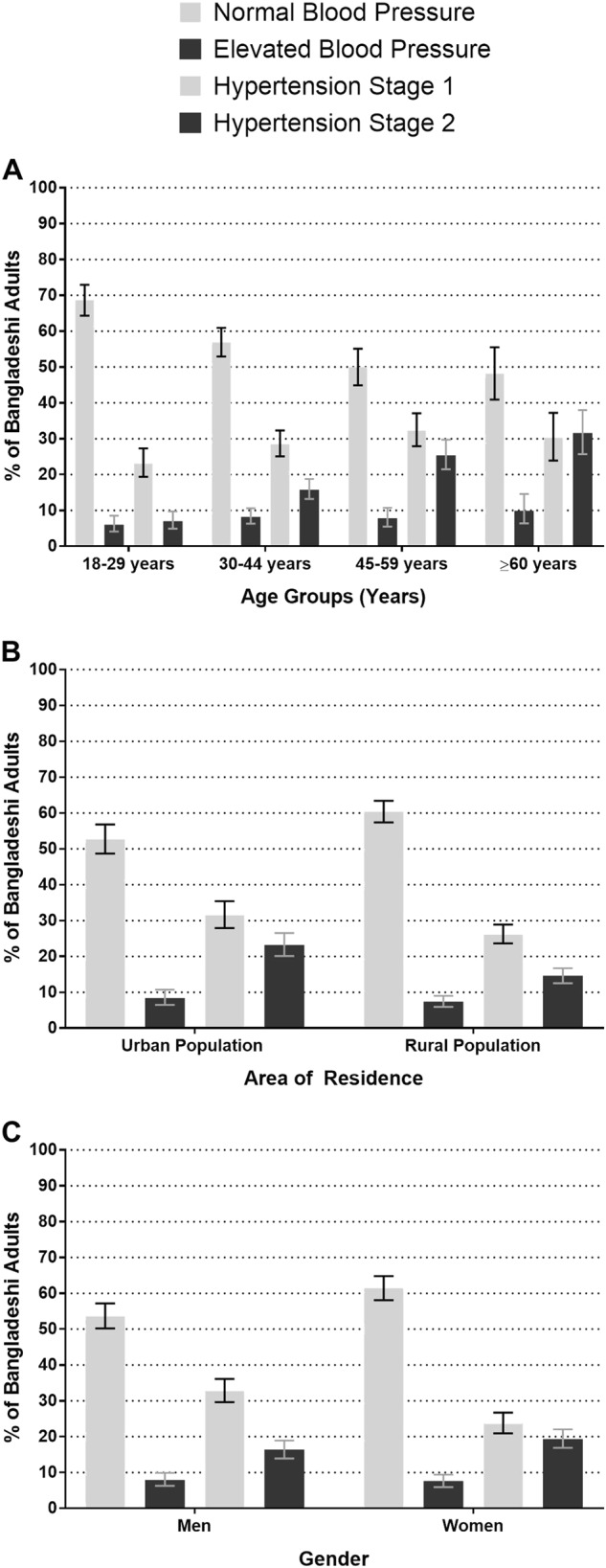
Prevalence of hypertension by stage as defined by the 2017 ACC/AHA Hypertension Clinical Practice Guidelines among Bangladeshi adults aged 18 years and above by (**a**) age group, **b** area of residence, and **c** sex, 2015 (*n* = 1843)

The 2017 ACC/AHA guidelines provides defined categories of hypertension to outline levels of severity (Figure 2). Fifty-seven percent of Bangladeshi adults had normal BP (<120 mmHg), and this proportion differed significantly across area (*p* = 0.002) and sex (*p* = 0.001). Similarly, hypertension stage 1 (SBP 130–139 mmHg or DBP 80–89 mmHg) was found among 28.2% adults, and also differed significantly across area of residence (*p* = 0.016) and sex (*p* < 0.001). Hypertension stage 2 (SBP≥140 mmHg or DBP≥90 mmHg) was found among, 17.9% of adults, and differed significantly across area of residence (*p* < 0.001), however, there was no significant difference across gender (*p* = 0.095).

### Risk factors of hypertension

We identified predictors for hypertension defined by the 2017 ACC/AHA guidelines and the JNC 7 guidelines separately to compare differences in risk factors (Table 2). Based on the 2017 ACC/AHA guidelines for hypertension, increasing age, low or moderate physical activity, past tobacco use, increasing BMI, abdominal obesity, and those with diabetes were more likely to have hypertension. The odds of hypertension were significantly highest among those aged 45–59 years (aOR 2.5, 95% CI: 1.8–3.3), and ≥ 60 years (aOR: 2.4 95% CI: 1.7–3.4) compared to individuals aged 18–29 years of age. Additionally, the odds of hypertension among those with low physical activity was 2.3 times the odds of those with vigorous physical activity (95% CI: 1.3–4.1). Past tobacco use increased the odds of hypertension by 60% in comparison to never tobacco usage. The odds of hypertension were significantly highest among those with a BMI over 30 or obesity (aOR: 2.6, 95%CI: 1.4–4.8) and overweight (aOR: 1.9, 95%CI: 1.4–2.5) when compared to normal BMI. Additionally, the odds of hypertension were 90% higher among those with abdominal obesity compared to those with a normal waist circumference. Diabetics had three times the odds of hypertension compared to non-diabetics (95% CI: 1.5–2.6). Being female (aOR: 0.5, 95% CI: 0.3–0.7) and living in a rural area (aOR: 0.8, 95% CI: 0.6–0.9) were both protective factors against hypertension.

**Table 2:** Predictors of Hypertension by two separate definitions among Bangladeshi adults, 2015 (*n* = 1843)

Total	2017 ACC/AHA hypertension definition			Joint National Committee (JNC) 7 Guidelines		
	Hypertension prevalence (%)^a^	95% CI	Adjusted OR (95% CI)^b^	Hypertension prevalence (%)^c^	95% CI	Adjusted OR (95% CI)^b^
	40.7	38.5–43.0		17.9	16.2–19.7	
Characteristic						
*Area*						
Urban	48.0	44.3–51.8	Ref.	23.2	20.1–26.5	Ref.
Rural	36.1	33.3–38.9	0.8 (0.6–0.9)	14.6	12.5–16.7	0.8 (0.6–1.0)
*Gender*						
Male	43.4	40.1–46.7	Ref.	16.4	14.0–18.9	Ref.
Female	38.3	35.2–41.4	0.5 (0.3–0.7)	19.3	16.9–22.0	0.9 (0.5–1.5)
*Age (years)*						
18–29	28.2	24.2–32.4	Ref.	7.1	4.9–9.7	Ref.
30–44	39.8	36.0–43.6	1.6 (1.2–2.1)	15.8	13.2–18.8	2.3 (1.5–3.6)
45–59	49.8	45.1–54.4	2.5 (1.8–3.3)	25.4	21.5–29.7	4.2 (2.7–6.5)
≥60	51.9	45.2–58. 5	2.4 (1.7–3.4)	31.6	25.7–38.0	4.7 (2.9–7.7)
*Occupation*						
Professional employment^d^	48.2	42.2–54.2	Ref.	19.6	15.2–24.8	Ref.
Unemployed/retired	64.3	53.9–73.7	1.3 (0.7–2.3)	44.9	34.8–55.3	1.8 (0.9–3.5)
Industrial worker/Day Laborer	32.5	28.3–36.9	0.9 (0.6–1.2)	8.9	6.5–11.8	0.7 (0.4–1.1)
Housewife	39.8	36.3–43.4	1.6 (1.0–2.6)	20.3	17.5–23.3	1.4 (0.8–2.5)
Other^e^	41.8	35.4–48.4	1.3 (0.9–1.9)	15.1	10.7–20.4	1.1 (0.7–1.9)
*Wealth index* ^f^						
1st Wealth quartile	38.6	33.9–43.5	Ref.	16.9	13.4–20.9	Ref.
2nd Wealth quartile	37.1	33.0–41.4	0.9 (0.7–1.2)	15.3	12.4–18.6	0.9 (0.6–1.3)
3rd Wealth quartile	44.3	39.5–49.1	1.2 (0.9–1.6)	19.3	15.7–23.3	1.2 (0.8–1.8)
4th Wealth quartile	43.5	38.9–48.2	0.9 (0.7–1.2)	20.6	16.9–24.6	0.9 (0.6–1.4)
*Physical activity*						
Vigorous	36.2	33.6–38.9	Ref.	13.5	11.7–15.6	Ref.
Moderate	48.2	43.6–52.7	1.4 (1.1–1.7)	24.5	20.9–28.6	1.4 (1.1–1.9)
Low	66.7	55.7–76.4	2.3 (1.3–4.1)	45.9	35.2–57.0	2.8 (1.5–5.3)
*Tobacco use* ^g^						
Never	39.1	35.8–42.4	Ref.	16.6	14.2–19.3	Ref.
Current use	40.4	37.1–43.4	1.1 (0.9–1.5)	18.0	15.5–20.8	1.6 (1.1–2.1)
Past use	53.2	44.6–61.7	1.6 (1.0–2.4)	25.2	18.2–33.2	1.8 (1.1–3.1)
*Body mass index* ^h^						
Normal (≤25)	34.9	32.5–37.5	Ref.	13.9	12.2–15.8	Ref.
Overweight (25.1–30)	60.4	54.9–65.8	1.9 (1.4–2.5)	29.9	24.9–35.2	1.5 (1.1–2.2)
Obese (>30)	71.0	58.8–81.3	2.6 (1.4–4.8)	46.4	34.3–58.8	2.7 (1.5–4.9)
*Waist cirumference (cm)*						
Normal (<90 cm M, <80 cm F)	32.9	30.4–35.6	Ref.	11.5	9.8–13.4	Ref.
Abdominally obese (≥90 cm M, ≥80 cm F)	59.1	54.9–63.3	1.9 (1.5–2.6)	33.0	29.1–37.1	2.4 (1.7–3.4)
Diabetes^i^						
No	38.6	36.3–40.9	Ref.	15.6	13.9–17.4	Ref.
Yes	78.2	68.9–85.8	3.0 (1.8–2.6)	57.4	47.2–67.2	3.4 (2.1–5.6)

*OR* odds ratio, *CI* confidence intervals, *Ref* referent category

^a^Defined based on the ACC/AHA 2017 Hypertension Clinical Guidelines of systolic blood pressure ≥ 130 mmHg and/or diastolic blood pressure ≥ 80 mmHg

^b^Model adjusted for all variables included in table: area, sex, age, occupation, wealth index, physical activity, tobacco use, body mass index, waist circumference, and diabetes diagnosis

^c^Defined based on the Joint National Committee (JNC) 7 Guidelines blood pressure ≥ 140 mmHg and/or diastolic blood pressure ≥ 90 mmHg

^d^Professional occupation includes: field staff, police officer, guard, doctor, engineer, professional, business man, desk job

^e^Other occupation includes: shop keeper, weavers, driver, student, beggar, cook, carpenter, tailor, migrant workers and fishermen

^f^Wealth index was calculated using principal component analysis using data collected on household ownership of the following items: electricity, flushable toilet, land phone, cell phone, television, radio, refrigerator, private car, motor cycle, washing machine, bicycle, sewing machine, almirah/wardrobe, table, bed, chair/bench, watch/clock, as well as, type of main material used to build their homes roof, walls and floor

^g^Includes both smokeless tobacco and smoke tobacco

^h^Body mass index (BMI) calculated by weight in kilogram divided by height in meter squared

^i^Diabetes is defined as a blood glucose level of ≥11.0 mmol/l

Based on the JNC 7 Guidelines, the risk factors for hypertension included increasing age, low or moderate physical activity, current and past tobacco use, increasing BMI, abdominal obesity, and having diabetes. The odds of hypertension were significantly highest among those aged ≥ 60 years (aOR: 4.7 95% CI: 2.9–7.7) compared to individuals aged 18–29 years. Both moderate and low physical activity increased the odds of hypertension by 1.4 (95% CI: 1.1–1.9) and 2.8 (95% CI: 1.5–5.3), respectively, when compared to the odds of vigorous physical activity. Current and past tobacco use increased the odds of hypertension by 60% and 80%, respectively, in comparison to never tobacco use. The odds of hypertension were significantly highest among those with a BMI over 30 or obesity (aOR: 2.7, 95% CI: 1.5–4.9). Additionally, the odds of hypertension among those with abdominal obesity was 2.4 times the odds of those with normal waist circumference (95% CI, 1.7–3.4). The odds of hypertension among diabetics were 3.4 times that of non-diabetics.

The only difference in risk factors identified was current tobacco use, which was identified as a predictor of hypertension based on the JNC 7 guidelines. Additionally, for the JNC 7’s definition of hypertension, female gender and rural area of residence were no longer significant protective factors against hypertension as observed for the 2017 ACC/AHA model.

### Treatment patterns of hypertension

**Fig. 3:**
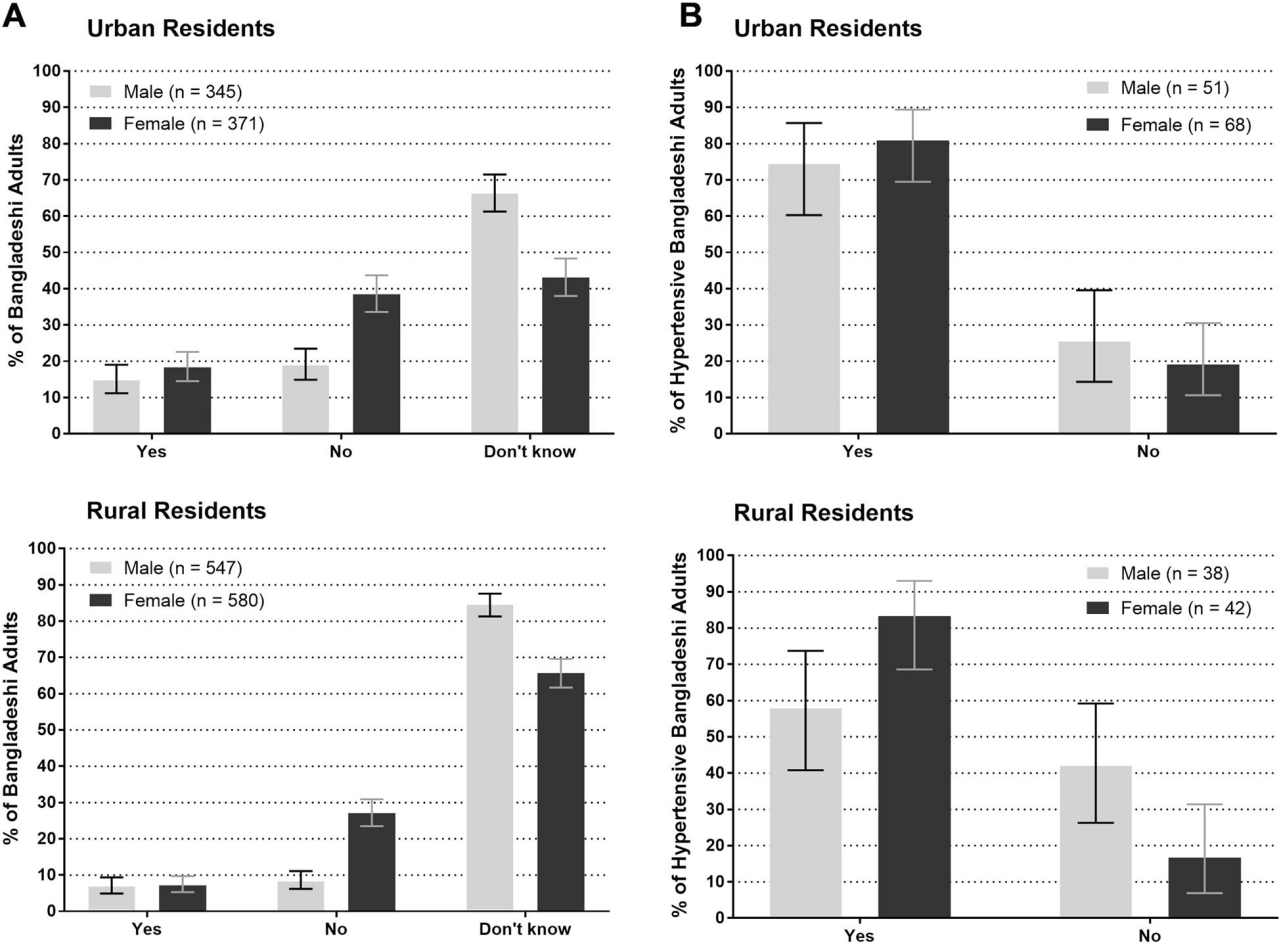
Bangladeshi adults aged 18 years and above (**a**) who have been diagnosed with hypertension by a health care worker, and **b** hypertensive adults who take medications to control their hypertension based on self-report, 2015

We recorded treatment history of hypertension as defined by JNC 7 guidelines (current cut-off points for hypertension in Bangladesh). One hundred and ninety-nine participants (10.8%) reported to have been previously diagnosed with hypertension by a health care provider. However, 26 of these individuals did not meet the JNC 7 definition of hypertension as they did not take medication to control their hypertension and their SBP was ≤140 or their DBP was ≤90 mmHg. Therefore, 52.4% of hypertensive adults, per the JNC 7’s definition, were previously diagnosed with the condition. The proportion of men and women who self-reported to be previously diagnosed with hypertension by a health care provider was higher among urban residents (men: 14.8%; women: 18.3%) than among rural residents (men: 6.9%; women: 7.2%) (Fig. 3a). However, overall, the large majority (66.9%) reported they did not know if they had been previously diagnosed with hypertension; this proportion was higher among rural residents (74.9%) than urban residents (54.3%).

Among participants previously diagnosed with hypertension (*n* = 199), 75.4% reported to take medication to control their hypertension. Among urban participants, women more frequently (80.9%) self-reported to take hypertension medication than men (74.5%) (Fig. 3b). Among rural participants, the proportion of women who did not take medication to control their hypertension was also higher (83.3%) than men (57.9%) (Fig. 3b).

Although about three-quarters (*n* = 150) of hypertensive participants reported to take medication to control their hypertension, 61% continued to have high BP levels indicating uncontrolled hypertension at study measurement. Among the 61% with uncontrolled high BP, the average systolic BP was 148.3 mmHg (SD:16.4) and average diastolic BP was 94.1 mmHg (SD: 9.7). Additionally, their average age was 49.9 years; the average age among men was 48.4 years and among females was 50.8 years.

**Fig. 4:**
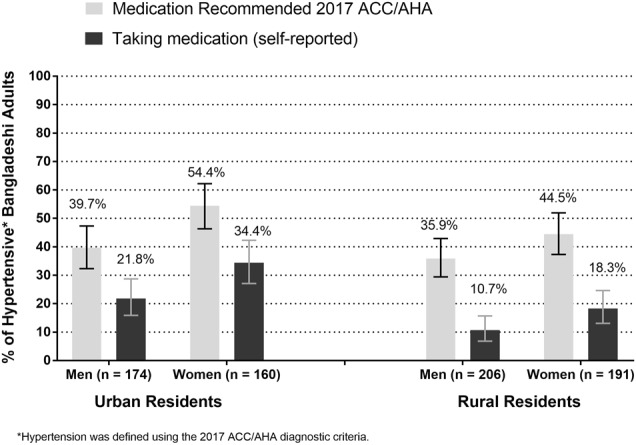
Prevalence of hypertensive Bangladeshi adults (≥18 years) recommended for pharmacological treatment according to 2017 ACC/AHA Hypertension Clinical Practice Guidelines compared to those who take hypertensive medications

Figure 4 presents prevalence of hypertensive adults who reported to take medication to control their hypertension as defined by the JNC7 guidelines, compared to those recommended for pharmacological treatment according to 2017 ACC/AHA. Per the 2017 ACC/AHA guidelines, 39.7% and 54.4% of hypertensive urban men and women are recommended for pharmacological treatment, respectively.

## Discussion

In this nationally representative survey of Bangladeshi adults aged ≥18 years, we found the prevalence of hypertension was 17.9% based on existing guidelines (JNC 7). This prevalence increased to 40.7% when the 2017 ACC/AHA criteria for diagnosis of hypertension was applied, indicating a 22.8% increase in prevalence. Predictors of increased odds of hypertension did not change across the two definitions of hypertension, however, being female and residing in a rural area were significant protective factors against hypertension per the 2017 ACC/AHA hypertension definition only. Overall, the Bangladeshi hypertensive adult population is under-treated, particularly the rural population. Using the existing guidelines, half of hypertensive adults were aware of their condition, and one-quarter were not taking medication to control their hypertension. With the new guidelines, a higher proportion of adults with high BP will be recommended for pharmacologic treatment to avoid subsequent cardiovascular disease outcomes [23].

The prevalence of hypertension among Bangladeshi adults on a national level has been previously studied [16, 24, 25]. Prior studies conducted with technical support of the WHO Country Office for Bangladesh found the prevalence of hypertension among adults aged ≥25 years to be 20% in 2010 [16], and 21% in 2013 [25]. In our study, the prevalence of hypertension was 17.9%, which is lower than previously documented. Our estimated prevalence of hypertension may be lower than prior studies as our study population was younger, starting at the age of 18 years. Using the 2017 ACC/AHA diagnostic criteria for hypertension, the prevalence of hypertension among Bangladeshi adults aged ≥18 years may rise to 40.7%. Using the latest population estimates from the BBS of adults aged ≥18 years [19], we can estimate the change in prevalence of hypertension would lead to an absolute increase from about 20.5 million adults (JNC 7 guidelines) to about 45.6 million adults (2017 HCPG guidelines). Although the increase in burden of hypertension may cause a strain on the health care system, readjusting the diagnostic criteria of hypertension to lower cut-off points following the 2017 ACC/AHA may lead to a strong focus on prevention of CVDs as hypertension will be diagnosed at an earlier stage [12, 26]. Timely access and availability of treatment for CVDs, such as cardiac stents, thrombolysis, or surgical interventions, is limited in Bangladesh; prevention is key to reducing the burden and mortality due to NCDs in a resource-limited setting [27]. However, availability of preventive treatment and access to primary care services through well-coordinated intervention strategies and efforts will need to be prioritized for successful roll-out of updated Bangladeshi guidelines [9]. Currently, the primary health care workforce in Bangladesh is not equipped to address the high increase in burden of hypertension in the adult population. Alternative strategies to improve primary care in Bangladesh, such as the use of community health workers [28], should be emphasized. Additionally, identification of those at an increased risk of developing hypertension will be key to appropriate allocation of resources and personnel, including community health workers.

Predictors of hypertension among Bangladeshi adults have been previously evaluated, however, here we present a comparison using the 2017 ACC/AHA and the current JNC 7 guidelines diagnostic criteria used in Bangladesh. For both definitions of hypertension, we find that increasing age, low physical activity, increasing BMI, abdominal obesity, and diabetes are predictors of having hypertension. Prior national surveys have identified these predictors among Bangladeshi adults [16, 29] and other low-income and middle-income countries. However, in our analysis, we identify two interesting points when evaluating predictors identified using the 2017 ACC/AHA criteria for diagnosis of hypertension; the first points is that the odds of developing hypertension among rural adults is 20% lower when compared to urban adults. Prior studies present conflicting results on the differences in hypertension prevalence among urban and rural residents of Bangladesh; national surveys have identified a higher burden of hypertension among urban residents compared to rural residents [29, 30] and also no differences in burden of hypertension between urban and rural residents [14]. Our second point of interest was that we found that women have 50% lower the odds of developing hypertension when compared to men. This finding is in contrast to prior studies which have found that women are at an increased risk of developing hypertension compared to men due to lifestyle factors, such as increased waist circumference and low physical activity [14]. Our findings may be of public health interest and implies men and urban residents should be prioritized as target populations for intervention to reduce the burden of hypertension in Bangladesh. However, as this is a descriptive exploratory analysis, further study is warranted to potentially replicate findings and inform future research questions.

Published in 2013, the National Guidelines for Management of Hypertension in Bangladesh were developed to provide country-specific guidelines for effective management of hypertension by primary care providers in a low-resource context [31]. The current Bangladeshi guidelines are written based on the JNC 7 guideline’s diagnostic criteria of hypertension. Bangladesh’s hypertension guidelines highly recommend the use of lifestyle modification or non-pharmacological management at all stages of hypertension as first-line treatment options due to the low resources available in the country. In fact, the algorithm for management of hypertension outlined in Bangladesh’s guidelines recommends initiating pharmacologic treatment when BP reads SBP 140–159 mmHg and/or DBP 90–99 mmHg only in the company of major risk factors, such as target organ involvement or diabetes. Pharmacologic intervention is immediately recommended for those to have SBP ≥ 160 mmHg and/or DBP ≥ 100, with 3–6-month follow-up and assessment. Based on the 2017 ACC/AHA guidelines, these cut-off points for pharmacologic treatment should be revisited. Although the guidelines may not be uniformly accepted by physicians in the United States [7], revision of Bangladeshi guidelines for hypertension management may lead to improved efforts towards prevention of subsequent CVDs. Recommendations should be adapted from the 2017 ACC/AHA as suitable for a low-resource setting context. The BP cut-off points for pharmacologic treatment initiation should also be re-evaluated to improve treatment recommendations for those at risk for developing CVDs.

The 2017 ACC/AHA guidelines strongly support nonpharmacologic interventions as primary and secondary approaches to lowering BP. Such interventions include, following a heart-healthy diet, such as the dietary approach to stop hypertension (DASH) diet, reduction in dietary sodium intake (<1500 mg/d or at least 1000 mg/d reduction), potassium supplementation (3500–5000 mg/d), physical activity increase (90–150 min per week), moderation of alcohol intake (≤2 drinks per day in men and ≤1 drink per day in women), and weight loss. Most of these interventions have been shown to reduce BP in randomized control trials by 5–10 mmHg [4, 32–34]. Additionally, tobacco use should be addressed in Bangladesh as we observed that 40% adults who use some form of tobacco had hypertension [25]. Despite an observed reduction in tobacco use in Bangladesh over the past decade [35], tobacco use continues to be a highly acceptable and common practice in Bangladesh, particularly among men [35]. Smokeless tobacco is also a highly common practice among adults in Bangladesh, and frequently also used in tandem with smoking tobacco in the form of cigarettes [36]. To reduce the use of tobacco products among Bangladeshi adults, tobacco control policies and targeted interventions should be developed and successfully implemented into practice.

Adherence to treatment should also be considered as a major factor in the decision to prescribe either lifestyle or pharmacological treatment to patients. In our study, we found that although 75% of hypertensive participants with known diagnosis reported to take medication, 61% of these hypertensive adults continued to have high BP. This may be due to poor adherence to treatment, which has been previously documented as a concern among adults both in urban and rural areas of Bangladesh [13–15]. Additionally, therapeutic inertia, or the failure of health-care providers to intensify or initiate therapy despite continued high BP, may be a major cause of observed uncontrolled hypertension as seen in prior studies [37]. Several factors have been found to be associated with poor adherence to hypertension treatment among rural adults only in Bangladesh including male gender and hypertension diagnosis by unqualified health providers or community health workers, in comparison to qualified physicians [13]. Emphasis on the importance of medication adherence should be prioritized in future training of community health workers, as they may not be adequately communicating its significance. Educational campaigns on the role of community health workers in the community may improve trust in these front-line workers among potential hypertension patients.

Our study has several strengths to be highlighted. Data collected was of a nationally representative sample, and included Bangladeshi adults aged 18 years or above. We present novel prevalence data of hypertension among adults aged 18 years and above, as prior studies have started their cohorts at older ages [16, 24, 25]. Our data will be necessary to measure the country’s progress towards a global target of the WHO’s NCD Global Monitoring Framework: a 25% relative reduction in the prevalence of raised BP [38] among persons aged 18+ years [38]. Additionally, we used WHO-recommended standardized methods to measure BP among our study population to limit the potential for measurement error.

In addition, our study has several limitations which should be considered. As our study design is cross-sectional we are unable to assess causality between the observed associations and hypertension. Questions regarding prior diagnosis of hypertension and treatment of hypertension are vulnerable to recall bias; however, when available, doctor’s prescription or medicine labels were checked. Physical activity may be misclassified due to recall bias; we assume there may be underreporting of low physical activity [22]. Additionally, we were unable to estimate the number of participants with high cardiovascular risk defined as a history of cardiovascular disease or 10-year predicted disease risk ≥10%. As such, we underestimated the number of participants who would be recommended for treatment based on the 2017 ACC/AHA, and were only able to include adults aged 65 years and above to those recommended for treatment based on JNC7. Future studies should be designed to estimate 10-year risk of CVD among Bangladeshi adults and also, include questions to assess family history of CVD and personal history of CVD.

## Conclusion

If adopted, the 2017 ACC/AHA guidelines may lead to an increase in burden of hypertension among Bangladeshi adults aged 18 years and above from about 18% to about 40%. In response to the potential doubling of the burden of hypertension, the Government of Bangladesh will need to take several steps to implement appropriate programs for prevention and treatment of this chronic disease. Preventive methods such as lifestyle changes and medication should be recommended by primary care providers in Bangladesh to avoid the future development of CVDs. National initiatives such as training community health workers to deliver primary care and implementing universal health coverage should be considered to curb the spread of hypertension and consequent CVDs in Bangladesh.

### Summary

#### What is known about the topic?

- Few national-based studies have been conducted in Bangladesh to assess the prevalence of hypertension in Bangladesh, and currently there is no relevant data available on adults aged 18–25 years.
- Risk factors for hypertension among Bangladeshi populations include high body mass index, age, area of residence, and gender.
- The majority of cases of hypertension in Bangladesh are untreated and undetected.

#### What this study adds?

- Here, we provide the first analysis of prevalence of hypertension using the American College of Cardiology and American Heart Association’s (ACC/AHA) 2017 Hypertension Clinical Practice Guidelines, in comparison to the existing guidelines, called Joint National Commission 7, guidelines among Bangladeshi adults aged 18 years and above.
- This study shows that with the adoption of the 2017 ACC/AHA guidelines, the prevalence of hypertension will be more than double in Bangladesh.
- National efforts to improve treatment options among hypertensive adults should be prioritized, as the Non-Communicable Disease (NCD) Control Program of the Government of Bangladesh prepares to respond to the potential increase in prevalence of hypertension and subsequent burden on the health care system.

## References

[1] A global brief on hypertension: silent killer, global public health crisis. Geneva, Switzerland: World Health Organization; 2013.

[2] Kearney PM, Whelton M, Reynolds K, Muntner P, Whelton PK, He J (2005). Global burden of hypertension: analysis of worldwide data. Lancet (London, England).

[3] Lim SS, Vos T, Flaxman AD, Danaei G, Shibuya K, Adair-Rohani H (2012). A comparative risk assessment of burden of disease and injury attributable to 67 risk factors and risk factor clusters in 21 regions, 1990-2010: a systematic analysis for the Global Burden of Disease Study 2010. Lancet (London, England).

[4] Whelton PK, Carey RM, Aronow WS, Casey DE, Jr., Collins KJ, Dennison Himmelfarb C, et al. 2017 ACC/AHA/AAPA/ABC/ACPM/AGS/APhA/ASH/ASPC/NMA/PCNA guideline for the prevention, detection, evaluation, and management of high blood pressure in adults: a report of the American College of Cardiology/American Heart Association Task Force on Clinical Practice Guidelines. Hypertension. 2017. 10.1161/HYP.0000000000000065.

[5] Greenland Philip, Peterson Eric (2017). The New 2017 ACC/AHA Guidelines “Up the Pressure” on Diagnosis and Treatment of Hypertension. JAMA.

[6] Cifu Adam S., Davis Andrew M. (2017). Prevention, Detection, Evaluation, and Management of High Blood Pressure in Adults. JAMA.

[7] Cohen JB, Townsend RR (2018). The ACC/AHA 2017 hypertension guidelines: both too much and not enough of a good thing?. Ann Intern Med.

[8] Nishimura RA, Otto CM, Bonow RO, Carabello BA, Erwin JP, Fleisher LA (2017). 2017 AHA/ACC focused update of the 2014 AHA/ACC guideline for the management of patients with valvular heart disease: a report of the American College of Cardiology/American Heart Association Task Force on Clinical Practice Guidelines. J Am Coll Cardiol.

[9] Al Mamun M, Rumana N, Pervin K, Azad MC, Shahana N, Choudhury SR (2016). Emerging burden of cardiovascular diseases in Bangladesh. J Atheroscler Thromb.

[10] Lozano R, Naghavi M, Foreman K, Lim S, Shibuya K, Aboyans V (2012). Global and regional mortality from 235 causes of death for 20 age groups in 1990 and 2010: a systematic analysis for the Global Burden of Disease Study 2010. Lancet (London, England).

[11] Forouzanfar MH, Liu P, Roth GA, Ng M, Biryukov S, Marczak L (2017). Global burden of hypertension and systolic blood pressure of at least 110 to 115 mm Hg, 1990–2015. JAMA.

[12] Ahsan Karar Zunaid, Alam Nurul, Kim Streatfield Peter (2009). Epidemiological transition in rural Bangladesh, 1986–2006. Global Health Action.

[13] Khanam MA, Lindeboom W, Koehlmoos TL, Alam DS, Niessen L, Milton AH (2014). Hypertension: adherence to treatment in rural Bangladesh—findings from a population-based study. Glob Health Action.

[14] Islam AK, Majumder AA (2012). Hypertension in Bangladesh: a review. Indian Heart J.

[15] Alam DS, Chowdhury MA, Siddiquee AT, Ahmed S, Niessen LW (2014). Awareness and control of hypertension in Bangladesh: follow-up of a hypertensive cohort. BMJ Open.

[16] Rahman Mujibur, Zaman M. Mostafa, Islam Jessica Yasmine, Chowdhury Jalil, Ahsan HAM Nazmul, Rahman Ridwanur, Hassan Mahtabuddin, Hossain Zakir, Alam Billal, Yasmin Rubina (2017). Prevalence, treatment patterns, and risk factors of hypertension and pre-hypertension among Bangladeshi adults. Journal of Human Hypertension.

[17] Haq SA, Ahmed S, Al-Quadir Z, Zaman MM, Moniruzzaman M, Khan MH. Prevalence of rheumatic disorders in Bangladeshi adults. Dhaka, Bangladesh: World Health Organization; 2015.

[18] Chronic disease and health promotion: STEP wise approach to surveillance (STEPS). Geneva: World Health Organization.

[19] Population and housing census 2001, updated in 2009. Dhaka, Bangladesh: Bangladesh Bureau of Statistics; 2009.

[20] Muntner P, Carey RM, Gidding S, Jones DW, Taler SJ, Wright JT (2018). Potential US population impact of the 2017 ACC/AHA high blood pressure guideline. Circulation.

[21] Global Physical Activity Questionnaire (GPAQ) analysis guide. World Health Organization 2017 http://www.who.int/ncds/surveillance/steps/STEPS_Manual.pdf.

[22] Moniruzzaman M, Mostafa Zaman M, Islalm MS, Ahasan HA, Kabir H, Yasmin R (2016). Physical activity levels in Bangladeshi adults: results from STEPS survey 2010. Public Health.

[23] Bakris George, Sorrentino Matthew (2018). Redefining Hypertension — Assessing the New Blood-Pressure Guidelines. New England Journal of Medicine.

[24] Rahman MM, Gilmour S, Akter S, Abe SK, Saito E, Shibuya K (2015). Prevalence and control of hypertension in Bangladesh: a multilevel analysis of a nationwide population-based survey. J Hypertens.

[25] Zaman MM, Bhuiyan MR, Karim MN, MoniruzZaman, Rahman MM, Akanda AW (2015). Clustering of non-communicable diseases risk factors in Bangladeshi adults: an analysis of STEPS survey 2013. BMC Public Health.

[26] Omran AR (1971). The epidemiologic transition. A theory of the epidemiology of population change. Milbank Mem Fund Q.

[27] Knowlton LM, Banguti P, Chackungal S, Chanthasiri T, Chao TE, Dahn B (2017). A geospatial evaluation of timely access to surgical care in seven countries. Bull World Health Organ.

[28] Chowdhury AM, Bhuiya A, Chowdhury ME, Rasheed S, Hussain Z, Chen LC (2013). The Bangladesh paradox: exceptional health achievement despite economic poverty. Lancet (London, England).

[29] Chowdhury MA, Uddin MJ, Haque MR, Ibrahimou B (2016). Hypertension among adults in Bangladesh: evidence from a national cross-sectional survey. BMC Cardiovasc Disord.

[30] Islam MZ, Akhtaruzzaman M, Lamberg-Allardt C (2004). Nutritional status of women in Bangladesh: comparison of energy intake and nutritional status of a low income rural group with a high income urban group. Asia Pac J Clin Nutr.

[31] National guidelines for management of hypertension in Bangladesh. Dhaka, Bangladesh: WHO/SEARO/Country Office for Bangladesh and DGHS, Director General of Health Affairs, Ministry of Health and Family Welfare; 2013. 82 pp.

[32] Cifu AS, Davis AM (2017). Prevention, detection, evaluation, and management of high blood pressure in adults. JAMA.

[33] Whelton Paul K., Carey Robert M., Aronow Wilbert S., Casey Donald E., Collins Karen J., Dennison Himmelfarb Cheryl, DePalma Sondra M., Gidding Samuel, Jamerson Kenneth A., Jones Daniel W., MacLaughlin Eric J., Muntner Paul, Ovbiagele Bruce, Smith Sidney C., Spencer Crystal C., Stafford Randall S., Taler Sandra J., Thomas Randal J., Williams Kim A., Williamson Jeff D., Wright Jackson T. (2018). 2017 ACC/AHA/AAPA/ABC/ACPM/AGS/APhA/ASH/ASPC/NMA/PCNA Guideline for the Prevention, Detection, Evaluation, and Management of High Blood Pressure in Adults. Journal of the American College of Cardiology.

[34] Sacks FM, Svetkey LP, Vollmer WM, Appel LJ, Bray GA, Harsha D (2001). Effects on blood pressure of reduced dietary sodium and the Dietary Approaches to Stop Hypertension (DASH) diet. DASH-Sodium Collaborative Research Group. N Engl J Med.

[35] Nargis N, Thompson ME, Fong GT, Driezen P, Hussain AK, Ruthbah UH (2015). Prevalence and patterns of tobacco use in Bangladesh from 2009 to 2012: evidence from International Tobacco Control (ITC) Study. PLoS One.

[36] Zaman MM, Bhuiyan MR, Huq SM, Rahman MM, Sinha DN, Fernando T (2014). Dual use of tobacco among Bangladeshi men. Indian J Cancer.

[37] Lebeau JP, Cadwallader JS, Aubin-Auger I, Mercier A, Pasquet T, Rusch E (2014). The concept and definition of therapeutic inertia in hypertension in primary care: a qualitative systematic review. BMC Fam Pract.

[38] NCD Global Monitoring Framework: World Health Organization. [cited 29 Sep 2017]. http://www.who.int/nmh/global_monitoring_framework/en/ (2017). Accessed date: February 21st, 2018.

